# N-Acetylcysteine Attenuates Ischemia-Reperfusion-Induced Apoptosis and Autophagy in Mouse Liver via Regulation of the ROS/JNK/Bcl-2 Pathway

**DOI:** 10.1371/journal.pone.0108855

**Published:** 2014-09-29

**Authors:** Chengfen Wang, Kan Chen, Yujing Xia, Weiqi Dai, Fan Wang, Miao Shen, Ping Cheng, Junshan Wang, Jie Lu, Yan Zhang, Jing Yang, Rong Zhu, Huawei Zhang, Jingjing Li, Yuanyuan Zheng, Yingqun Zhou, Chuanyong Guo

**Affiliations:** Department of Gastroenterology, Shanghai Tenth People's Hospital, Tongji University School of Medicine, Shanghai, China; Virginia Commonwealth University, United States of America

## Abstract

**Background:**

Hepatic ischemia–reperfusion injury (HIRI) remains a pivotal clinical problem after hemorrhagic shock, transplantation, and some types of toxic hepatic injury. Apoptosis and autophagy play important roles in cell death during HIRI. It is also known that N-acetylcysteine (NAC) has significant pharmacologic effects on HIRI including elimination of reactive oxygen species (ROS) and attenuation of hepatic apoptosis. However, the effects of NAC on HIRI-induced autophagy have not been reported. In this study, we evaluated the effects of NAC on autophagy and apoptosis in HIRI, and explored the possible mechanism involved.

**Methods:**

A mouse model of segmental (70%) hepatic warm ischemia was adopted to determine hepatic injury. NAC (150 mg/kg), a hepatoprotection agent, was administered before surgery. We hypothesized that the mechanism of NAC may involve the ROS/JNK/Bcl-2 pathway. We evaluated the expression of JNK, P-JNK, Bcl-2, Beclin 1 and LC3 by western blotting and immunohistochemical staining. Autophagosomes were evaluated by transmission electron microscopy (TEM).

**Results:**

We found that ALT, AST and pathological changes were significantly improved in the NAC group. Western blotting analysis showed that the expression levels of Beclin 1 and LC3 were significantly decreased in NAC-treated mice. In addition, JNK, p-JNK, Bax, TNF-α, NF-κB, IL2, IL6 and levels were also decreased in NAC-treated mice.

**Conclusion:**

NAC can prevent HIRI-induced autophagy and apoptosis by influencing the JNK signal pathway. The mechanism is likely to involve attenuation of JNK and p-JNK via scavenged ROS, an indirect increase in Bcl-2 level, and finally an alteration in the balance of Beclin 1 and Bcl-2.

## Introduction

Hepatic ischemia reperfusion injury (HIRI) was recognized as a main cause of pathological damage by Toledo-Pereyra et al. in 1975 during research on liver transplantation [1]. HIRI can be divided into warm ischemia reperfusion injury and cold-storage reperfusion injury [2], [3]. The former is clinically relevant in liver surgery, hypovolemic shock, liver transplantation, some forms of toxic liver injury and Budd-Chiari syndrome [4]. The latter occurs during organ preservation before transplantation [5], [6]. It is recognized that an excessive inflammatory response is an important mechanism of ischemia reperfusion injury [7]. The activation of Kupffer cells and neutrophils contribute to the formation of reactive oxygen [8], [9], [10]. There is still no explicit mechanism of HIRI, Therefore, the possible mechanism of HIRI and how to reduce ischemia reperfusion damage are important research issues. Thus, identification of an effective novel therapeutic is urgently required.

Ischemia-reperfusion injury is a complex pathophysiological process which involves Kupffer cell activation, the production of reactive oxygen species (ROS), the release of chemokines and cytokines, mitochondrial permeability transition, neutrophil recruitment and the pH paradox [11], [12]. The main pathophysiological changes in HIRI are inflammatory cells infiltration, proinflammatory factors release and eventually hepatocyte death [13]. Two mechanisms are involved in the process of hepatocyte death. One is necrosis, a type of non-programmed cell death, which is characterized by swelling of cells and organelles, membrane breakdown causing release of cell contents and activation of inflammatory factors. The other mechanism is apoptosis, named type I programmed cell death, which is characterized by cell shrinkage, DNA programmed degradation and chromatin condensation [14], [15]. The mechanism of apoptosis initiation involves many stimuli including TNFα, Fas ligand and DNA damage. These stimuli lead to activation of the caspase family (cysteine-aspartate proteases) which can break cells into many small vesicles called apoptotic bodies [16], [17]. The mitochondrial pathway is also involved in another mechanism of apoptosis, which is closely related to the Bcl-2 family. The Bcl-2 family includes both anti-apoptotic proteins such as Bcl-2 and Bcl-xL and pro-apoptotic proteins such as Bax, Bad and Bak [18], [19]. In addition, apoptosis and necrosis are not completely independent, as they may share downstream pathways and signals. Thus, the phenomenon of necrapoptosis occurs in many pathophysiological conditions.

Autophagy is another important form of cell death, and is an intracellular degradation process where lysosomes degrade proteins, cellular organelles and invading microbes [20]. There are three different types of autophagy: macroautophagy, chaperone-mediated autophagy (CMA), and microautophagy [21]. At basal levels, autophagy contributes to cellular homeostasis. When nutrients are depleted and stresses occur in cells, autophagy can be further induced. Regulators of autophagy include the target of rapamycin (TOR), TOR kinase, 5′-AMP-activated protein kinase (AMPK), eukaryotic initiation factor 2α (eIF2α), inositol-trisphosphate (IP3) and c-Jun-N-terminal kinase [22], [23]. Beclin 1, UVRAG, Vps34, Vps15 and Bif-1 play an important role in vesicle nucleation [24]. In addition, the anti-apoptotic proteins Bcl-2 and Bcl-xL bind to the pro-autophagy protein, leading to an inversely proportional relationship [25], [26]. Light chain 3 (LC3) is a marker of autophagy, which contributes to the formation of autophagy vesicles during vesicle nucleation. LC3 and phosphatidylethanolamine (PE) conjugate and result in the formation of non-soluble LC3 (LC3 II). The presence of LC3 II allows autophagy to be detected by biochemical or microscopy techniques [27], [28]. In brief, autophagy can promote cell survival by digesting free fatty acids and misfolded proteins. However, auto-lysosomes may degrade cellular membrane lipids, which can activate enzymes and proinflammatory cytokines. Thus, autophagy is a double-edged sword in terms of cell survival. Autophagy plays a significant role in the liver which is a dynamic organ.

HIRI is a complex process involving necrosis, apoptosis, and autophagy. Preconditioning and pharmacologic interventions can increase the resistance of liver cells to HIRI. Therefore, an investigation of the possible mechanisms and novel treatment of HIRI is meaningful. In this study, the therapeutic effects and possible mechanisms of N-acetylcysteine (NAC) in an ischemia–reperfusion model were determined. NAC has been used as an antioxidant for the treatment of many clinical diseases such as doxorubicin-induced cardiotoxicity, acetaminophen (paracetamol) intoxication, bronchitis, heavy metal toxicity, and hepatic encephalopathy [29]. NAC is a source of glutathione (GSH) and sulfhydryl groups, and a scavenger of free radicals due to its interaction with ROS. NAC is known as an “antioxidant” in experimental models. It has been reported to prevent apoptosis and increase cell survival by activating the extracellular signal regulated kinase pathway [30]. Treatment of HIRI with NAC impedes NF-kappa B activity and ROS expression. How NAC affects the JNK signal pathway has not yet been fully elucidated.

## Materials and Methods

### 2.1 Reagents

N-acetylcysteine (NAC) was purchased from Sigma-Aldrich (St. Louis, MO, USA). The following antibodies were used in this research: anti-TNF-α (0.1 ug/ml; Santa Cruz, CA, USA), anti-IL-6 and anti-IL-2 (both from Proteintech, CA, and USA), anti-Bax, anti-Bcl-2, anti-Beclin 1, anti-LC3, anti-JNK, anti-p-JNK, anti-NF-κB (all from Cell Signal Technology, USA). ROS Fluorescent Probe-DHE was purchased from Vigorous. (Beijing, China).

### 2.2 Animals

Male Balb/c mice (6-8 weeks old, 22±2 g) were purchased from Shanghai SLAC Laboratory Animal Co. Ltd (Shanghai, China). They were fasted in plastic cages which were maintained at 26°Cand 55% humidity. The mice had free access to food and water. The study and the experimental design were approved by the Ethics Committee of Shanghai Tongji University.

### 2.3 Experimental design

The mice were randomly divided into three groups: group I (saline only) included 18 mice which were injected with saline via the tail vein before laparotomy without I/R, group II (IR model) included 18 mice which were injected with saline via the tail vein 1 h before they underwent segmental (70%) hepatic warm ischemia for 40 min, and group III (NAC + IR) which included 18 mice which were injected with NAC (dissolved in saline) via the tail vein 1 h before they underwent IR for 40 min. Six mice from each of the three groups were selected and were sacrificed 6 h, 12 h and 24 h after IR. At the end of the experiment, serum and liver tissue samples were obtained from each mouse and stored for further analysis.

### 2.4 Analysis of liver enzymes

Alanine aminotransferase (ALT) and aspartate aminotransferase (AST) levels in the collected serum were analyzed using an automated clinical analyzer (OLYMPUS AU1000; Olympus, Tokyo, Japan).

### 2.5 Histopathology

Mouse liver tissues (left lobe) were collected, stored in 4% paraformaldehyde and embedded in paraffin. Sections (5 µm thick) were stained with hematoxylin-eosin (H&E) and observed by light microscopy.

### 2.6 Immunohistochemical staining

The sections were heated at 67°C for 30 min and then dewaxed in dimethylbenzene. The sections were then dehydrated in a concentration gradient of alcohol and pretreated with microwave heat-induced epitope retrieval. These sections were incubated with antibodies against Bcl-2, Bax, Beclin 1, LC3, JNK and p-JNK at 1∶100 dilution for 24 h at 4°C and then with a secondary antibody at 1∶50 dilution for 1 h at 28°C. All the antibodies were diluted with Tris-buffered saline (TBS), and 2% bovine serum albumin (BSA). Finally, the sections were stained by diaminobenzidine (DAB) and then observed using a digital camera (Olympus) combined with a light microscope at ×200 magnification.

### 2.7 Western blotting analysis

Proteins were extracted from the liver tissues by grinding with protease inhibitors and stored at -80°C. The proteins were incubated in boiling water for 10 min before the experiment. The protein samples were separated by 12.5% sodium dodecyl sulfate polyacrylamide gel electrophoresis (SDS-PAGE) and transferred onto a Polyvinylidene Fluoride (PVDF) membrane. The membranes were incubated with 5% nonfat milk for 1 h, and then incubated with antibodies against TNF-α, IL-6, IL-2, Bax, Bcl-2, Beclin 1, LC3, JNK, p-JNK, NF-κB at 4°C for 24 h. The following day, the samples were incubated with the secondary antibody at 37°C for 30 min. The results were determined using the Odyssey Two-Color Infrared Laser Imaging System.

### 2.8 TUNEL staining

TUNEL staining was carried out after the sections were dewaxed, dehydrated and rehydrated. After that the sections were re-dyed with hematoxylin. The sections were then placed under a microscope to observe TUNEL positive cells and hepatocytes.

### 2.9 ROS assay

Fresh liver tissues of mice were fixed in 4% paraformaldehyde on ice for 1 h. The fixed tissues were washed three times by PBS for 10 min on ice before they were dehydrated overnight in 30% sucrose at 4°C. Then, the tissues were infiltrated with OCT (SAKURA, USA) for 2 h, and stored at −80°C. Sections (5 µm) were cut with a freezing microtome. The sections dried at room temperature for 5 min, and then were washed three times by PBS for 5 min. The sections were incubated with ROS Fluorescent Probe-DHE (50 µM, diluted by PBS) for 90 min. After, the sections were washed three times by PBS for 10 min. Then sections were sealed with quenching agent and observed with fluorescence microscopy. All the process need to avoid light.

### 2.10 Transmission Electron Microscopy (TEM)

Following collection of liver tissues from the sacrificed mice, a fraction of the left liver lobe was placed in 4% glutaraldehyde and post fixed in 1% OsO4. The cells were observed by TEM (JEOL, JEM 1230).

### 2.11 Reverse transcription-polymerase chain reaction (RT-PCR) and SYBR Green Real-time PCR

RNA was extracted from liver tissues using TRIzol Reagent (Takara, Shiga, Japan). Total RNA was reversed transcribed into cDNA using the SYBR Premix Ex Taq kit (TaKaRa Biotechnology China). We used SYBR Green quantitative RT-PCR to determine the expression of the target genes. The primers used for this experiment were as Table 1.

**Table 1:** 

Gene		Primer Sequence(5'→3')
**Bax**	Forward	AGACAGGGGCCTTTTTGCTAC
	Reverse	AATTCGCCGGAGACACTCG
**Bcl-2**	Forward	GCTACCGTCGTCGTGACTTCGC
	Reverse	CCCCACCGAACTCAAAGAAGG
**Beclin 1**	Forward	ATGGAGGGGTCTAAGGCGTC
	Reverse	TGGGCTGTGGTAAGTAATGGA
**LC3**	Forward	GACCGCTGTAAGGAGGTGC
	Reverse	AGAAGCCGAAGGTTTCTTGGG
**NF-κB**	Forward	ATGGCAGACGATGATCCCTAC
	Reverse	CGGATCGAAATCCCCTCTGTT
**IL-6**	Forward	CTGCAAGAGACTTCCATCCAG
	Reverse	AGTGGTATAGACAGGTCTGTTGG
**TNF-α**	Forward	CAGGCGGTGCCTATGTCTC
	Reverse	CGATCACCCCGAAGTTCAGTAG

### 2.12 Statistical analysis

Measurement data were expressed as mean ± SD. Statistical differences between different groups were determined via one-way ANOVA and Tukey's post hoc test for multiple comparisons when F was significant. All statistical analyses were performed using SPSS 17.0 statistical software.

## Results

### 3.1 NAC protects rats from hepatic ischemia-reperfusion injury

**Figure 1:**
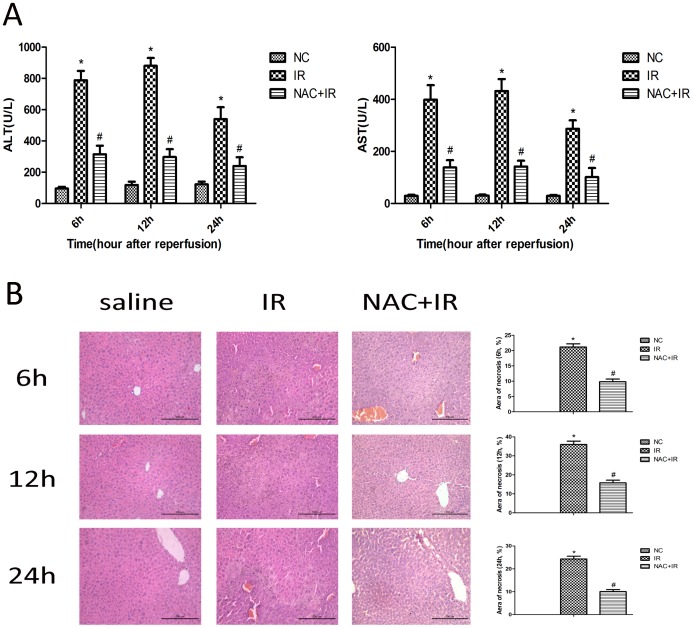
Effect of NAC on hepatic ischemia-reperfusion injury. The ischemia-reperfusion and sham-operated mice were pretreated with NAC (150 mg/kg) or saline. (A) Serum AST and ALT levels were measured at 6, 12 and 24 h after reperfusion of the three groups. Data represent means (SD) (n = 6 mice per time point per group). *p<0.05 for saline VS saline + IR, # p<0.05 for saline +IR VS IR + NAC (150 mg/kg). (B) Photomicrographs of representative livers collected 6, 12 and 24 h after reperfusion, stained with hematoxylin and eosin (H&E), ×200 magnification. The necrotic areas were analyzed with Image-pro Plus 6.0, indicating there existed statistical significant among different groups [n = 6, *p<0.05 for saline VS saline + IR, # p<0.05 for saline +IR VS IR + NAC (150 mg/kg)].

Segmental (70%) hepatic warm ischemia for 40 min led to a rise in aminotransferase (AST 410±112 U/L, ALT 850±226 U/L) when compared to the NAC-pretreatment (150 mg/kg) groups (AST 130±12 U/L, ALT 300±24 U/L) (Figure 1A). As, expected, control groups showed much lower levels of aminotransferase than either IR or NAC + IR (AST 53±17 U/L, ALT 120±28 U/L). The pathological changes of the hepatic tissues from the three groups after hematoxylin and eosin staining (H&E) are shown in Fig 1B. It can be obviously observed that the use of NAC significantly alleviated hepatocyte necrosis after ischemia-reperfusion when compared to IR groups. In the IR groups, we can observed disordered lobular structure, marked hepatocyte necrosis, and polymorphonuclear cell infiltration. Control groups showed intact structure of hepatic tissues. These results demonstrated that NAC pretreatment ameliorated HIRI-induced liver dysfunction.

### 3.2 NAC inhibited apoptosis in hepatic ischemia-reperfusion injury

**Figure 2:**
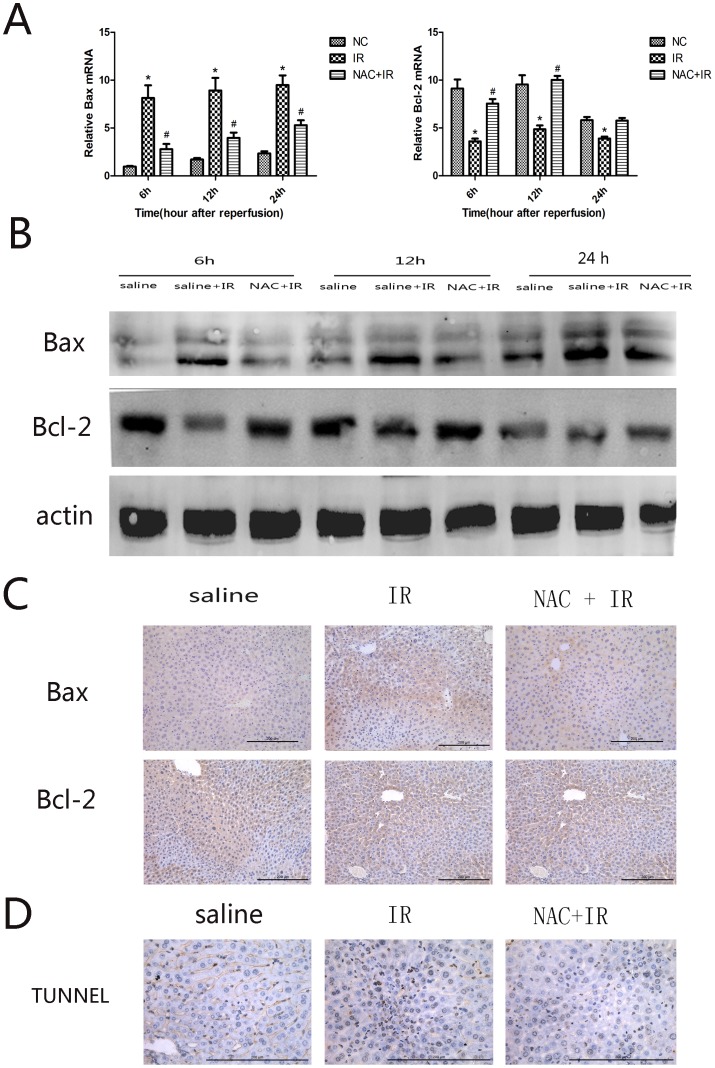
Effect of NAC on regulation of apoptosis. (A) The expression of Bax and Bcl-2 on cDNA levels were detected by real time PCR. ^*^ *p*<0.05 for saline VS saline + IR, ^#^ *p*<0.05 for saline +IR VS IR + NAC (150 mg/kg). (B) The expression of Bax and Bcl-2 on protein levels were detected with western blot. (C) The expression of Bax and Bcl-2 on protein levels were detected by immunohistochemistry staining in hepatic tissues at 12 h (×200 magnification). (D) TUNEL staining of hepatic tissues in three groups at 12 h to detect apoptotic cells (×400 magnification).

Previous studies have reported evidence for the participation of apoptotic cell death in the hepatic ischemia-reperfusion. The Bcl-2 family is known to regulate apoptosis. Thus, to study the possible protective mechanism of NAC in HIRI, we detected the levels of Bcl-2 and Bax. As shown in Fig 2A, we can observe the changes of Bcl-2 and Bax at cDNA levels between three groups. The levels of the anti-apoptotic protein, Bcl-2, significantly increased in the NAC + IR group compared with IR group (*P*<0.005). The levels of Bax, a pro-apoptotic protein, were significantly increased in the IR group compared with NAC + IR group (*P*<0.005). And we examined the Bcl-2 family at the protein level (Figure 2B) to confirm the positive effects of NAC. It can be obviously observed that increase expression of Bax and decrease expression of Bcl-2 in the NAC pretreatment group when compared with the IR group. And both of Bcl-2 and Bax at low expression in the control group. In addition, there existed similar results in immunohistochemistry (Fig 2C). The apoptotic cells were measured with TUNEL staining (Figure2D). Numerous TUNEL-positive cells were observed in the model groups, whereas NAC pretreatment significantly reduced the number of TUNEL-positive cells (IR VS NAC + IR, *P*<0.005). These results demonstrated that NAC has a beneficial effect on HIRI-induced apoptosis.

### 3.3 NAC inhibited autophagic programmed cell death in hepatic ischemia-reperfusion injury

**Figure 3:**
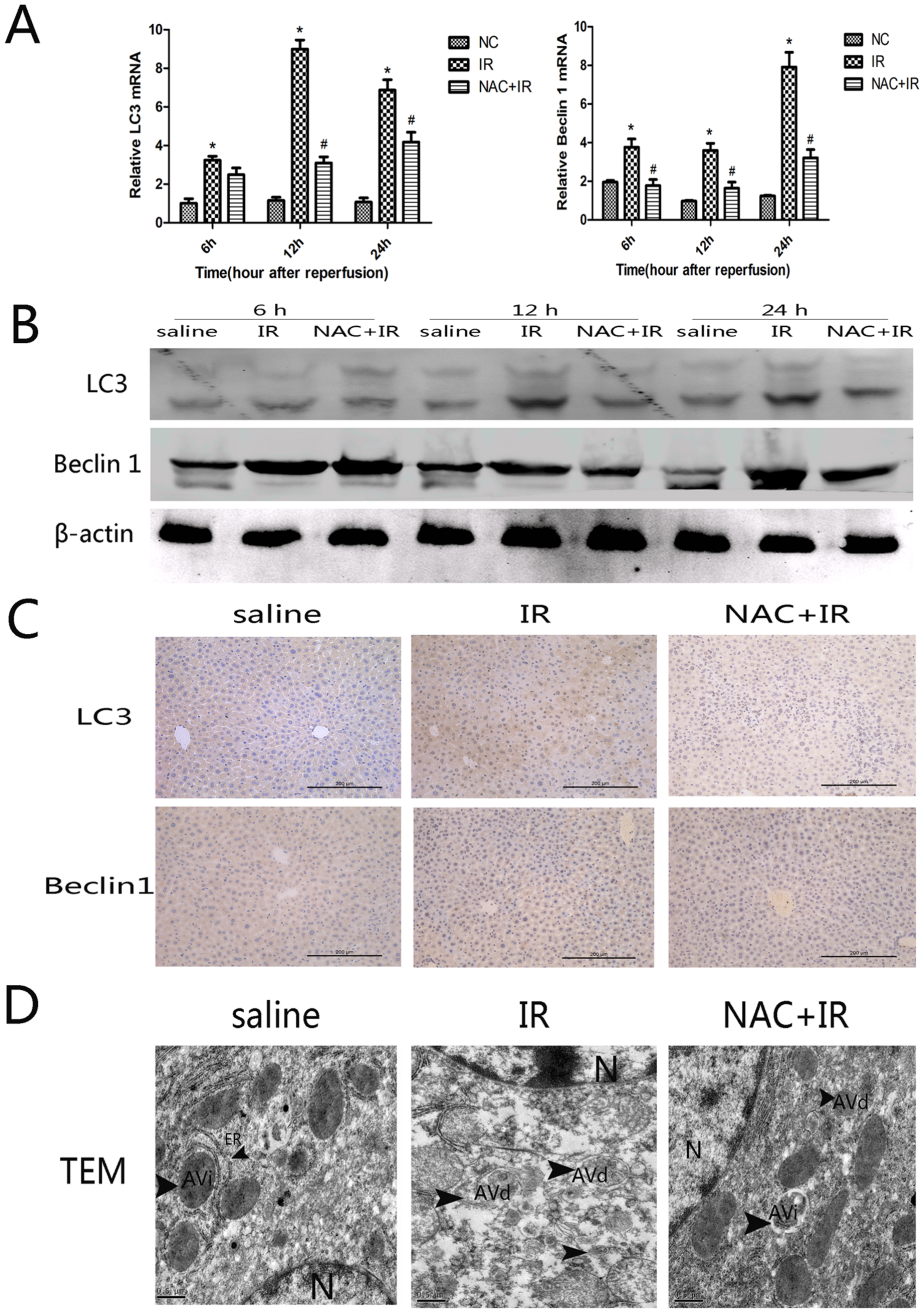
Effect of NAC on regulation of autophagy. (A) The expression of Bclin 1 and LC3 on cDNA levels were detected by real time PCR. ^*^ *p*<0.05 for saline VS saline + IR, ^#^ *p*<0.05 for saline +IR VS IR + NAC (150 mg/kg). (B) The expression of Bclin 1 and LC3 on protein levels were detected with western blot. (C) The expression of Bclin 1 and LC3 on protein levels were detected by immunohistochemistry staining in hepatic tissues at 12 h (×200 magnification). (D) Morphology of autophagosomes in hepatocytes at 12 h detected by electron microscopy (×20000 magnification). Initial autophagic vacuole (AVi) containing a mitochondrion, endoplasmic reticulum membranes, and ribosomes (arrowheads). Degradative autophagic vacuole (AVd) is found to have degradation of contents (arrowheads).

During the evaluation of the effects of NAC on autophagy, the levels of LC3 and Beclin 1 were measured by both immunohistochemistry and western blot. Beclin 1 and LC3 play a significant role in regulating autophagy. In this study, we detected the changes of Beclin 1 and LC3 both in cDNA and protein levels with real-time PCR (Fig 3A) and western blotting (Fig 3B). There were significantly increased in the IR group compared with the control and NAC + IR groups (*P*<0.005). And this result is concordant with the changes measured by immunohistochemistry (Fig 3C). It is also important to note the quantity and morphology of autophagosomes during autophagy. Furthermore, we used TEM to determine the ultrastructure of hepatic cells. TEM of HIRI showed a significant ultrastructural morphological change in hepatic cell necrosis. The IR group showed uneven nuclear chromatin, with partial agglutination and altered organelle structure, partial damage in the cytoplasm, numerous mitochondria in the cytoplasm, with damaged mitochondrial cristae and the organelle structure was not obvious. In addition, lysosomes and autophagosomes were obviously increased (Figure 3D). In the NAC + IR group, hepatic nuclear chromatin was homogeneous with a small number of agglutinations and rich organelles in the cytoplasm, and the structure of the mitochondria still had integrity (Figure 3D).

### 3.4 NAC inhibited the JNK signal pathway in hepatic ischemia-reperfusion injury

**Figure 4:**
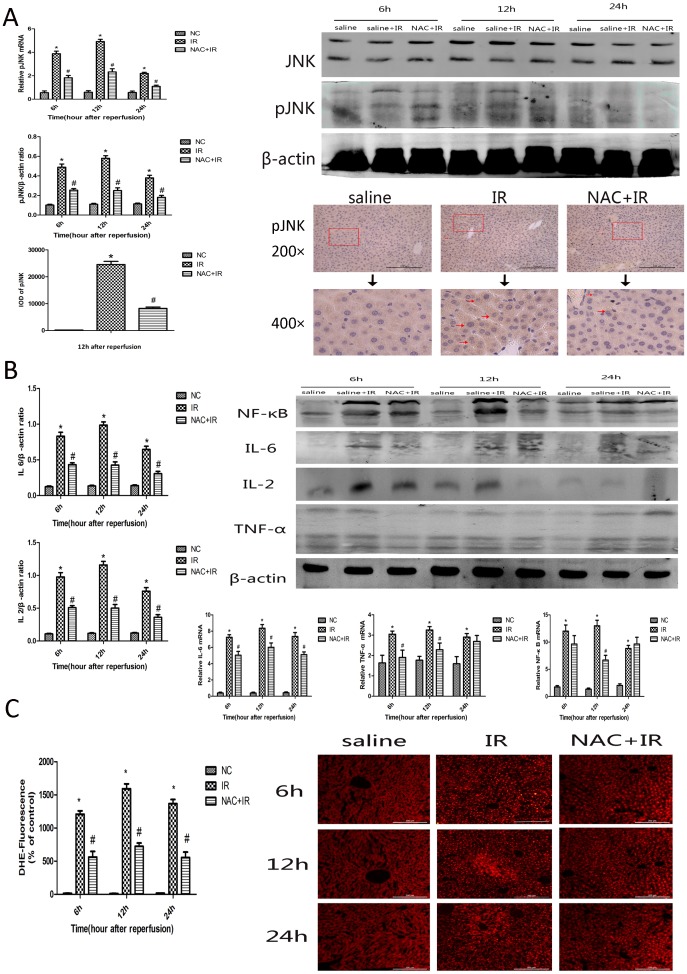
Effect of NAC on regulation of JNK pathway, cytokines release and ROS generation. (A) The expression of p-JNK and JNK on protein level was detected by western blot. The result of western blot were analyzed with quantity one [n = 3, *p<0.05 for saline VS saline + IR, # p<0.05 for saline +IR VS IR + NAC (150 mg/kg)]. The expression of p-JNK in hepatic tissue of different groups were shown by immunohistochemistry at 12 h (×200 magnification). The IOD of p-JNK in cytoplasm was analyzed by Image-Pro Plus 6.0. Date are showed as mean± SD [n = 3, *p<0.05 for saline VS saline + IR, # p<0.05 for saline +IR VS IR + NAC (150 mg/kg)]. The representative positive cells were indicated with red arrows. (B) The expression of NF-κB, IL-6, TNF-αon cDNA level were detected by real time PCR. *p<0.05 for saline VS saline + IR, # p<0.05 for saline +IR VS IR + NAC (150 mg/kg). And the expression of NF-κB, IL-6, TNF-α on protein level was detected by western blot. The result of the western blot were analyzed with quantity one [n = 3, *p<0.05 for saline VS saline + IR, # p<0.05 for saline +IR VS IR + NAC (150 mg/kg)]. (C) The generation of ROS was detected with ROS Fluorescent Probe-DHE. And ROS were measured and statistical analysis in three random vision fields by Image-Pro plus 6.0.

Previous studies proved that JNK is an important regulator of cell death including apoptosis and autophagy. To determine whether JNK signaling pathway is involved in hepatic ischemia-reperfusion induced cell death, we detected the phosphorylation of JNK in protein level in three groups respectively by western blotting (F 4A). As shown in Fig 4A, HIRI increased the activation of JNK phosphorylation. In the IR group, there was obvious up-regulation of p-JNK compared with the control group and NAC + IR group (*P*<0.005) in protein levels. And this result is concordant with the changes measured by immunohistochemistry (Fig 4A). These results suggest that NAC protects the liver from HIRI through inhibition of the p-JNK signaling pathway.

### 3.5 NAC attenuated cytokines and intracellular ROS generation induced by ischemia–reperfusion

Ischemia–reperfusion injury primarily occurs in hypoxic organs. The imbalance between pro- and antioxidants in the hypoxic organ leads to oxidative stress. Oxidative stress can increase the release of cytokines by activating inflammatory cells. Further activated neutrophils can generate ROS. To explore the effect of NAC attenuation on cytokines and ROS, we detected cytokines (TNF-a, IL-2, IL-6, and NF-κB) both at the cDNA and protein levels. As shown in Figure 4B, there was significantly increased expression of cytokines in the IR group compared with the NAC pretreatment group. There was also a decrease in cytokine expression in the control group. We also measured ROS generation by a ROS Fluorescent Probe-DHE in mice hepatic tissues. The proportion of ROS and normal areas were measured. Statistical analysis in three random visual fields was performed using Image-Pro plus version 6.0 software. As shown in Figure 4C, significantly increased ROS generation was observed in the IR group, compared with the control group. The level of ROS was significantly decreased in the NAC + IR group (p<0.005). Thus, pretreatment with NAC attenuated ischemia–reperfusion-induced ROS generation, cytokines release, and ROS generation.

## Discussion

Hepatic ischemia–reperfusion injury remains a significant clinical problem following transplantation. HIRI is a phenomenon which depends on cellular damage in a hypoxic organ, and destroys the restoration of oxygen exchange [2]. Hypoxia increases reactive oxygen species (ROS) in injured cells. ROS then causes apoptosis and autophagy [31], [32]. However, the exact mechanism involved in hepatic ischemia–reperfusion injury has not yet been fully described. We demonstrated that HIRI involves histopathologic changes, an increase of cytokines, as well as apoptotic cells, autophagosomes, and activation of the pro-apoptotic Bcl-2 family and the JNK pathway. Moreover, ischemia–reperfusion triggers cellular ROS [33]. The protective effect of NAC may involve the JNK pathway, as we observed significant changes in phosphorylation levels of JNK between the IR and NAC + IR groups.

NAC is a thiol and major precursor of L-cysteine [30]. NAC has been used in clinical practice for several years to treat clinical disorders such as stable angina pectoris, ischemia-reperfusion cardiac injury, doxorubicin-induced cardiotoxicity, acute respiratory distress syndrome and radio-contrast-induced nephropathy [34]. NAC has significant pharmacological effects on oxidative injury and is known as a source of sulfhydryl groups and a scavenger of free radicals [27]. It was demonstrated that NAC may protect cells through other mechanisms besides scavenging radicals. Our research showed that NAC protected against HIRI by attenuating hepatic apoptosis and autophagy. NAC decreased the levels of liver enzymes and attenuated histopathologic changes such as cellular swelling and cellular necrosis. Compared with the IR group, TUNEL-positive cells in the NAC + IR group were obviously reduced. This indicated that NAC can attenuate apoptosis caused by ischemia-reperfusion injury. It is thought that the Bcl-2 family plays a key role in the process of apoptosis. This family includes anti-apoptotic proteins such as Bcl-2 and Bcl-xL and pro-apoptotic proteins such as Bax and Bak [18], [19]. In this study, we observed a clear decrease in Bcl-2 and an increase in Bax in the IR group compared with the NAC + IR and control groups. We suggest that NAC decreases apoptosis by stabilizing the balance between Bcl-2 and Bax. In addition, IR activated the JNK pathway, as shown by a significant increase in the levels of phosphor-JNK. The results of our study indicate that the possible mechanism of apoptosis and autophagy inhibition by NAC may involve the JNK pathway.

Apoptotic and autophagic cell death are the two main forms of hepatic ischemia-reperfusion-induced cell death [35]. Autophagy also plays a key role in the hepatic pathophysiological disorders caused by liver ischemia-reperfusion injury. NAC may be a potential treatment option for HIRI. During our evaluation of the effects of NAC on autophagy in HIRI, we detected the expression of Beclin 1 and LC3II. The levels of Beclin 1 and LC3II in group III (NAC + IR) were significantly decreased. Compared to group III (NAC+ IR) and group I (model control), the levels of Beclin1 and LC3II in group II (IR) were significantly increased. These results indicate that NAC can improve HIRI-induced autophagy and liver injury, which suggests that inhibition of autophagy may be a new pathway in NAC therapy for improving liver injury. It has been reported that ROS through the JNK pathway regulates cell death [36], [37]. This pathway may be involved in many cellular events related to cell death including apoptosis and autophagy. In the present study, NAC partly attenuated apoptosis by Bcl-xL and Bcl-2, which act as important downstream factors of the JNK pathway [25]. In addition, ROS activate the JNK pathway, as shown by the increased levels of phosphorylated JNK. The findings in this study suggest that NAC may first reduce the levels of JNK and phosphorylated JNK through ROS scavenging, then causing Bcl-2 to increase. The effects of the latter interfere with Beclin 1 during the process of autophagy. The conclusion of our present research that inhibiting autophagy alleviated hepatic damage in ischemia-reperfusion induced liver injury models is in consistent with our previous studies [38], [39]. Therefore, we suggest that suppression of autophagy may be a potential therapeutic method in ischemia-reperfusion liver injury.

These findings demonstrated that NAC ameliorated apoptosis and autophagy which occurred during hepatic ischemia-reperfusion. These results indicate that the ROS/JNK/Bcl-2/Beclin 1 pathway may act as an intersection between apoptosis and autophagy. Recently, many studies have indicated that JNK is a mediator of autophagy, and demonstrated that JNK contributes to autophagic cell death via phosphorylation of Bcl-2 [40], [41]. Phosphorylation of Bcl-2 can lead to Bcl-2 separating from Beclin 1, thereby alleviating the inhibitory effect on Beclin 1 [42], [43]. Hence, activation of JNK can alter the balance between Bcl-2 and Beclin 1. This may be the mechanism of action of JNK in the regulation of autophagy.

In conclusion, we demonstrated that ischemia–reperfusion-induced apoptosis and autophagy occurs through activation of the JNK/Bcl-2 pathway. Hepatic protection by NAC is mediated by ROS scavenging and targeting of the JNK pathway via the JNK/Bcl-2 pathway. Numerous studies have suggested that there may be a common signal pathway between autophagy and apoptosis, and together with our findings, this indicates that the ROS/JNK/Bcl-2 pathway may play a key role in the cross-talk between autophagy and apoptosis. Finally, these results provide insight into the mechanism of HIRI and propose a potential clinical treatment for NAC in HIRI.
